# A Potential Paradigm Shift in Managing Huntington’s Disease? A Meta-Analysis of the Recent Advances in Symptomatic Treatment and Gene Therapy

**DOI:** 10.1192/j.eurpsy.2025.1538

**Published:** 2025-08-26

**Authors:** A. Agrawal, R. Walwaikar, J. Parmar, A. A. Pillai, A. A. Kumar, F. Sheikh

**Affiliations:** 1 Humanitas University, Milan, Italy; 2 SSPM Medical College and Lifetime Hospital, Sindhudurg; 3 Government Medical College, Amritsar; 4 GMERS Medical College, Vadnagar, India; 5 Greater manchester mental health trust, Manchester, United Kingdom

## Abstract

**Introduction:**

Huntington’s Disease (HD) is a serious inherited neurodegenerative disorder characterized by a progressive decline in motor and cognitive functions, along with neuropsychiatric symptoms. The pathophysiology involves extended CAG repeats in the huntingtin (HTT) gene, leading to the toxic accumulation of protein aggregates and subsequent neurodegeneration. Current treatments for HD solely provide symptomatic relief and are merely adjunctive, with no effect on disease progression. Novel gene editing technologies, such as CRISPR on the other hand, directly target mutant huntingtin at either the DNA or RNA level, and may potentially delay or halt the progression of HD through their disease-modifying effects.

**Objectives:**

This review is aimed at critically evaluating the recent advances in gene therapy and symptomatic treatment approaches, focussing on both current treatments as well as first-generation human and preclinical studies targeting HD. The review also seeks to assess the rationale for these studies, the mechanisms of action involved, and their broader implications for clinical practice and patient care.

**Methods:**

A systematic review of the recent literature on drug-based and non-drug-based HD treatment approaches, including symptomatic and advanced gene-editing strategies, was conducted. Data pooling was carried out with random-effects models to provide calculated standardized mean differences (SMDs) and 95% confidence intervals (CIs). The review included a comprehensive investigation of strategies such as CRISPR-Cas9, prime editing, antisense oligonucleotides, and RNA interference, yielding promising data with prudent clinical utility.

**Results:**

Eighteen studies with a total of 2,152 participants were analyzed. A significant reduction in motor manifestations compared to controls was recorded with Tetrabenazine and Deutetrabenazine use, with an SMD of -0.65 (p < 0.001). Antidepressant supplementation showed promising effects on psychiatric symptoms, achieving an SMD of -0.70 (p < 0.001). Non-drug-related treatments moderately improved participants’ quality of life, as illustrated by an SMD of 0.58 (p < 0.001). In gene therapy-related interventions, CRISPR-Cas9 and prime editing approaches showcased a significant reduction in the levels of mutant huntingtin protein, with a mean of -1.25 (p < 0.001). Additionally, antisense oligonucleotides provided encouraging results, eliciting a dose-dependent reduction in RNA levels, with a mean of -0.85 (p < 0.001).

**Conclusions:**

Combining innovative symptomatic treatments with novel gene-editing technology marks a potential paradigm shift in managing Huntington’s Disease. While symptomatic therapies are paramount to ensure optimal patient comfort, recent advances in gene therapy research hold tremendous potential in modifying and curbing disease progression.

**Disclosure of Interest:**

None Declared

